# *Borrelia valaisiana* in Cerebrospinal Fluid

**DOI:** 10.3201/eid1009.030439

**Published:** 2004-09

**Authors:** Eudoxia Diza, Anna Papa, Eleni Vezyri, Stefanos Tsounis, Ioannis Milonas, Antonis Antoniadis

**Affiliations:** * Aristotle University of Thessaloniki, Thessaloniki, Greece

**Keywords:** letter, Borrelia valaisiana, neuroborrelliosis, PCR

**To the Editor:** Lyme borreliosis is the most common tickborne human disease in the Northern Hemisphere. The incidence of the disease is not the same throughout Europe; in southern Europe, the incidence ranges from 43% in Croatia to 1.1% in Greece. Suspected borreliosis cases have been reported in Greece, none were confirmed. *Ixodes ricinus*, the principal tick vector of *Borrelia burgdorferi* in Europe, is found in northern Greece. A low prevalence of *B. burgdorferi* antibodies was found in healthy persons in Greece (*1**,**2*); a frequency of 7.3% was found in arthritis patients (*1*), while a frequency of 16.9% was found in patients with neurologic disorders (E. Diza, unpub. data).

Polymerase chain reaction (PCR) has been used to detect *B. burgdorferi* DNA in humans and to determine genospecies (*3*). Isolates found in the United States have constituted a homogeneous group. In Europe, five different genospecies from the original *B. burgdorferi*, now called *burgdorferi sensu lato* complex, have been described: *B. burgdorferi sensu stricto*, *B. garinii*, *B. afzelii, B. valaisiana*, and *B. lusitaniae*. Pathogenicity for humans remains uncertain for *B. valaisiana* and *B. lusitaniae* (*4*).

Neuroborreliosis, the most serious manifestation of disseminated Lyme disease, has become the most frequently recognized arthropodborne infection of the nervous system in the United States and Europe. *B. garinii*, *B. afzelii*, and *B. burgdorferi sensu stricto* are confirmed causes of neuroborreliosis (*5*); however, *B. valaisiana* has not been isolated from cerebrspinal fluid (CSF) until this report.

We report the genetic detection of *B. valaisiana* in the CSF of a 61-year-old man with a history of spastic paraparesis, which is strong clinical evidence of advanced neuroborreliosis. Symptoms, mainly difficulty in walking, began approximately 10 years earlier, with a slow progressive course of neuroborreliosis. His medical history showed an unidentified sexually transmitted disease in 1982, an undefined episode of arthritis in the lower limbs in 1990, and a nonspecific rash in the genitals in 1995. The patient lived in South Africa from 1961 to 1997 and visited Thassos Island in northern Greece every year. The neurologic examination demonstrated an intense pyramidal spasticity in the lower limbs and moderate weakness (Medical Research Council grade 3) of the proximal muscles. Serial magnetic resonance imaging (MRI) of the brain showed small hyperintensities in the periventricular area on T2-weighted images; MRI of the spinal cord showed no abnormalities. Multiple sclerosis, B12 deficiency, human T-cell lymphotrophic virus-1 infection, structural inflammatory lesions of the spinal cord, motor neuron disease, and hereditary spastic paraplegia have been excluded. The patient was treated occasionally with intravenous penicillin G, as well as with corticosteroids, but no clinical improvement was achieved. Venereal disease reaction level was negative and all tests for syphilis in CSF were negative.

DNA was extracted from CSF, and a region of the chromosomal flagellin gene of *B. burgdorferi* was amplified by nested PCR (*3*). *B. afzelii* (VS461) DNA was used as a positive control. All precautions were taken to avoid contamination. The amplified PCR product was sequenced, and the sequence (Th1) was deposited in GenBank with the accession no. AY270021. Phylogenetic analysis showed that strain Th1 was clustering with strains belonging to *B. valaisiana* genomic group. Specifically, a nucleotide difference of 0.38% was observed among Th1 and isolates Ku10 and To76 (accession no. AYO83505 and AYO83504, respectively), which belong to *B. valaisiana* genomic group and were isolated from *ricinus* in Sweden (*6*). A genetic difference of 0.77% was observed between Th1 and *B. valaisiana* strain Tr29 (accession no. ABO91805) isolated from *I. ricinus* in Turkey (*7*), while the genetic difference between Th1 and *B. burgdorferi* (X15661) was much greater, 6.83%.

This report is the first of genetic detection of *B. valaisiana* in CSF, which indicates a probable association of this genospecies with disease in humans. *B. valaisiana* has been isolated from *I. ricinus* ticks collected from vegetation and from ticks engorged on birds, in several European countries, including Turkey (*7*). The pathogenic capabilities of *B. valaisiana* are still uncertain; it has been detected by PCR and restriction fragment length polymorphism analysis in skin biopsy specimens from two erythema migrans patients and from patients with mixed infection (erythema migrans and acrodermatitis chronica atrophicans) (*4*). Indirect evidence suggests that *B. valaisiana* is involved in some chronic clinical manifestations (*8*).

Borreliosis is difficult to diagnose by serologic evaluation and Western blot interpretation. In our patient, no intrathecal antibodies were produced to support clinical suspicion of disease. The low antibody titers could be attributed to antigenic variation between *B. valaisiana* and *B. burgdorferi sensu stricto*, which was used as antigen because no commercial kit is specific for *B. valaisiana*. Differences between the strain causing infection and the antigen may play a role in the false-negative results (*9*). The low antibody response in our patient could be caused by antimicrobial drugs and corticosteroid medication.

The high homology of the nucleotide sequence from our patient and respective *B. valaisiana* sequences from other European countries suggests that he likely was infected in Greece. The status of Lyme disease in southern Africa is unknown, but *Ixodes* spp. ticks have been found there, and preliminary evidence indicates that the disease may occur in humans in South Africa (*10*).

We detected *B. valaisiana* DNA in CSF of a patient with slow progressive spastic paraparesis, which suggests that this microorganism might be the causative agent of the disease. Nucleotide sequence information of *Borrelia* strains from clinical cases and ticks from different countries will elucidate the molecular epidemiology of the disease.
